# Efficacy assessment of mesenchymal stem cell transplantation for burn wounds in animals: a systematic review

**DOI:** 10.1186/s13287-020-01879-1

**Published:** 2020-08-28

**Authors:** Hanxiao Yi, Yang Wang, Zhen Yang, Zhiqin Xie

**Affiliations:** 1 The First Affiliated Hospital of Nanchang University, No. 17, Yongwai Zhengjie, Nanchang, JiangXi Province China; 2 Spine Surgery, Third Affiliated Hospital of Sun-Yat Sen University, No. 600, Tianhe Road, Tianhe District, Guangzhou, Guangdong Province China

**Keywords:** Burn wound, Stem cell therapy, Animals, Efficacy, Meta-analysis

## Abstract

**Background:**

Clinically, severe burns remain one of the most challenging issues, but an ideal treatment is yet absent. Our purpose is to compare the efficacy of stem cell therapy in a preclinical model of burn wound healing.

**Methods:**

Research reports on mesenchymal stem cells (MSCs) for burn wound healing were retrieved from 5 databases: PubMed, Embase, MEDLINE, Web of Science, and the Cochrane Library. The primary outcomes reported in this article include the un-healing rate of the wound area, the closure rate, and the wound area. Secondary outcomes included CD-31, vascular density, interleukin (IL)-10, thickness of eschar tissue, vascular endothelial growth factor (VEGF), and white blood cell count. Finally, a subgroup analysis was conducted to explore heterogeneity that potentially impacted the primary outcomes. A fixed-effects model with a 95% confidence interval (CI) was performed when no significant heterogeneity existed. Otherwise, a random-effects model was used. All data analysis was conducted by using Engauge Digitizer 10.8 and R software.

**Results:**

Twenty eligible articles were finally included in the analysis. Stem cell therapy greatly improved the closure rate (2.00, 95% CI 0.52 to 3.48, *p* = 0.008) and compromised the wound area (− 2.36; 95% CI − 4.90 to 0.18; *p* = 0.069) rather than the un-healing rate of the wound area (− 11.10, 95% CI − 32.97 to 10.78, *p* = 0.320). Though *p* was 0.069, there was a trend toward shrinkage of the burn wound area after stem cell therapy. Vascular density (4.69; 95% CI 0.06 to 9.31; *p* = 0.047) and thickness of eschar tissue (6.56, 95% CI 1.15 to 11.98, *p* = 0.017) were also discovered to be significantly improved in the burn site of stem cell-treated animals. Moreover, we observed that animals in the stem cell group had an increased white blood cell count (0.84, 95% CI 0.01 to 1.66, *p* = 0.047) 5 days post treatment. Other indicators, such as VEGF (*p* = 0.381), CD-31 (*p* = 0.335) and IL-10 (*p* = 0.567), were not significantly impacted.

**Conclusions:**

Despite limited data from preclinical trials, this meta-analysis suggests that stem cell therapy is curative in decreasing the burn wound area and provides some insights into future clinical studies of stem cell therapy for burns.

## Introduction

According to the World Health Organization (WHO), an estimated 265,000 people worldwide annually die from burns, and even nonfatal burns are a major cause of illness [1, 2]. Burns are classified into three degrees: first degree (the superficial layer of the epidermis), second degree (dermis), and third degree (full-thickness skin, even reaching the muscle and bone). Burns are the consequence of chemicals [3], irradiation [4], heat [5], and electricity [6] and potentially lead to dehydration, electrolyte imbalance, infections, and even death. Severe burns are a devastating injury that affects almost all organs and causes severe morbidity and mortality [7], and no ideal treatments for severe burns have been recommended.

Existing clinical management protocols include improved resuscitation and treatment of inhalation injuries (for patients with respiratory injuries), early debridement and closure of wounds, appropriate infection control, and metabolic support [2, 8, 9]. However, significant challenges remain. At the onset of the loss of skin integrity, this injury can result in fragile protective competence of the first physical barrier, blood loss, severe pain, and subsequent infections, especially during the process of healing, which is both time and nutrient consuming. For patients with extensive burns and limited donor skin, skin grafts are sometimes reticulated and expanded to achieve greater coverage. However, mesh grafts may take several weeks to mature, and the cosmetic results are often unsatisfactory to patients. In addition, complications such as infections or skin contracture may occur, leading to additional treatments and a prolonged healing process [10, 11]. Therefore, new treatments are urgently needed.

Currently, mesenchymal stem cells (MSCs) are widely reported to involve many pathophysiological processes [12–14] and have a curative effect in treating various diseases [15–17]. MSCs, which can differentiate into epidermal cells and skin appendages (sebaceous 1and sweat glands), can be isolated and amplified from the umbilical cord, bone marrow, fat, and other tissues and have recently been reported to decrease burn the wound area in rats and mice [14, 18]. Despite the multipotency of stem cells, an increasing number of studies have shown that the efficacy of MSCs primarily depends on the secretion of a variety of factors, such as vascular endothelial growth factor (VEGF), epidermal growth factor (EGF), fibroblast growth factor (FGF), insulin-like growth factor (IGF), platelet-derived factor growth factor (PDGF), transforming growth factor beta (TGF beta), IL-4, IL-6, and IL-10, which play an important role in angiogenesis, cell recruitment, immune regulation, and wound healing, rather than the stemness of cells [19].

Currently, a series of clinical trials (1 allogenic MSC, 1 cadaveric MSC, 1 bone marrow MSC) and a case-control prospective study (bone marrow MSC = 20, umbilical cord-MSC = 20, EE&G = 20) investigating the efficacy and safety of stem cell therapy have been completed. The results demonstrate that MSCs can effectively promote burn wound healing and are considered safe and effective, with great therapeutic potential for patients with severe burns [20–23]. However, to date, there has been no systematic and comprehensive preclinical analysis of stem cell therapy for burn wounds, and the integration of these preclinical data can offset knowledge gaps that may affect the future application of stem cells. Therefore, we conducted a systematic review and meta-analysis of animal studies of the use of MSCs for treating burn wounds.

## Methods and materials

### Search strategy

The items of this meta-analysis were reported according to the Preferred Reporting Items for Systematic Review and Meta-Analysis (PRISMA) [24] (see Additional file 1).

We searched the PubMed, Embase, MEDLINE, Web of Science, and Cochrane Library databases systematically and comprehensively (from onset to April 2020). To obtain as many articles as possible, the search strategy “((mesenchymal stem cells) OR MSCs) AND burn” was used in our search, and the species was set as “other animals” rather than “human beings” (see Additional file 2). In addition, other studies that may have been eligible were manually identified by references or other reviews related to this topic.

### Selection criteria

The primary inclusion criteria were the use of stem cells through the administration of exogenous stem cells to promote post-hyperthermia wound healing and skin regeneration after burn injury. Stem cells are referred to as pluripotent and multipotent progenitor cells with the ability to self-renew and differentiate into organ- or tissue-specific cells. In summary, studies employing stem cells from bone marrow, umbilical cord, and fat and reporting any aspects of burn wounds were included in the analysis.

The exclusion criteria were the use of embryonic stem cells (ESCs), progenitor cells (epithelial and dermal stem cells), and programmed stem cells. Nonthermal burns (such as chemical and electricity burns), injury to organs and mucosa other than skin (such as the esophagus and respiratory tract), and old burns were excluded. Studies that reported unexpected outcomes and outcomes of humans were eliminated. Studies that used self-control were excluded as well.

### Data extraction

From the included studies, we extracted the following data: (a) identity: author and year of publication, design; (b) animals for each study: number of animals, species, age, burn model, region, stem cell type; (c) treatment: dosage, administration, timing (post burn). Animal experiments differ from randomized clinical trials (RCTs) and retrospective studies from humans, as there are no suitable assessment strategies. Thus, a quality assessment of each study was not conducted.

The data used for analysis were independently analyzed by two authors (Yi Hanxiao and Wang Yang). If a contradiction occurred, the original article was rechecked to confirm the accuracy of the data.

### Types of outcome measures

The following indicators were used to evaluate the efficacy of MSCs in treating burn wounds: closure rate and un-healing rate of the wound area, and wound area. The closure rate and un-healing rate of the wound area were expressed as percentages of the area, while the wound area was the actual area (mm^2^) reported in the original articles and this meta-analysis. CD-31 and vascular density were used to assess the status of angiogenesis by immunohistochemical staining, expressed as the average optical density (AOD) and number of vascular rings, respectively. VEGF and IL-10 were detected by reverse transcription-polymerase chain reaction (RT-QPCR) and enzyme-linked immunosorbent assay (ELISA), respectively. Other indicators, such as white blood cell count and eschar tissue thickness, were also evaluated.

### Statistical analysis

This meta-analysis was conducted using R software 3.63 (University of Auckland, New Zealand). All data evaluated in this article were continuous data and are expressed as the standardized mean difference (SMD) with corresponding 95% CI to eliminate the influence from units and measures. The chi-squared value test and inconsistency index (*I*^2^) were used to assess the heterogeneity across each study. A random-effects model was used to analyze the data if a value of *p* < 0. 05 or *I*^2^ > 50%, which was considered to have significant heterogeneity. Otherwise, a fixed-effects model was used. Subgroup analysis was used to find potential sources of heterogeneity. A leave-one-out meta-analysis and meta-regression were conducted to explore potential heterogeneities. Publication bias was tested by Egger’s test *t* with R software (University of Auckland, New Zealand)

## Results

### Study screen

**Fig. 1:**
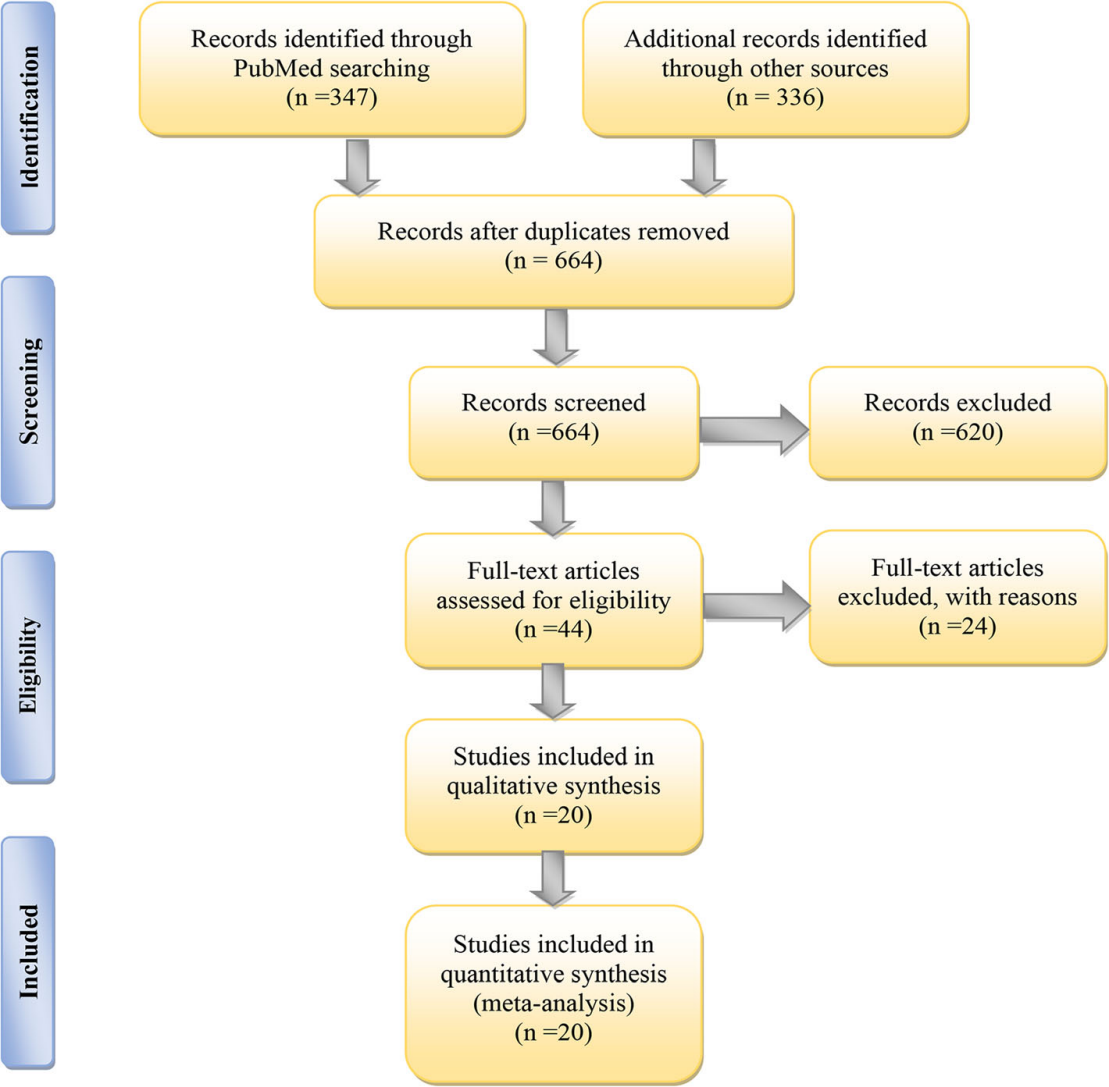
Flowchart of the article screening process

According to our search strategy, 683 articles were finally retrieved from PubMed, MEDLINE, Cochrane Library, Web of Science, and Embase databases. After elimination of duplicates, 664 articles were retained. Articles were further screened by browsing their titles and abstracts; 620 articles were excluded as irrelevant to the topic, and a total of 44 articles were determined for full-text review. Following careful review, 22 articles were removed as a result of the absence of anticipated outcomes associated with burn wounds. Eventually, 20 studies with parallel groups were included in this meta-analysis (Fig. 1).

### Study characteristics

The baseline characteristics of all studies are shown in Table 1. Published studies from 2003 to 2019, with 5 studies from China, 5 studies from the USA, 2 studies from Iran, 2 studies from Taiwan, and 1 each from Russian, Ireland, Turkey, Japan, and New Orleans, and Pakistan. The sample size ranged from 3 to 74. Most of the studies were conducted on rats and mice (18 out of 20), 2 of which were conducted on pigs. The studied cell types included bone MSCs (BMSCs, *n* = 6), adipose-derived MSCs (ADMSCs, *n* = 10), and umbilical cord MSCs (UC-MSCs, *n* = 4). The cell dosage ranging from 10^4^ to 10^6^ cells/wound was used immediately or 9 days post treatment. In 16 studies (80%), local injection and tail injection were performed, and in 3 studies (15%), stem cell-treated sterile dressings were applied. Other items, such as the objective of the study, species, age, and burn model, were also included.

**Table 1:** Characteristics of included studies

Author	Year	Region	Total	No. of wounds		Species/strain	Gender/weight(g)	Age	Model	Cell type	Dosage	AD	Timing (post burn)
				MSC	Con								
Xue	2013	China	60	30	30	Mice/ICR	M/NA	8–10 W	Partial thickness (3–5% TBSA), 90 °C water for 3 s	H-BMSCs	1 × 10^6^ cells/wound	Local injection	Immediately
Karimi	2014	Iran	18	9	9	Mice/Balb/c	M/40	NA	Full thickness, a 96 °C needle for 8 s	M-ADSCs	1 × 10^6^ cells/wound	Local injection	Immediately
Loder	2015	USA	10	5	5	Mice/C57BL/6	M/NA	26–28 W	Partial thickness (30% TBSA), 60 °C water for 17 s	Syngenic M- ADSCs	1 × 10^6^ cells/wound	Local injection	24 h
Bliley	2016	USA	24	12	12	Mice/athymic nude	F/NA	7–9 W	Full thiness, a 70 °C brass stamp for 10s	H-ADSCs	6.8 × 10^6^ cells/wound	Local injection	24 h
Shumakov	2003	Russian	30	20	10	Rat/Wistar	M/(300–350) g	Adult	Full thiness (18–20% TBSA), a 97.7 °C metal plate for 8 s	Auto/Allogenic R-BMSCs	2 × 10^6^ cells/wound	NA	48 h
Singer*	2013	USA	20	20	20	Rat/Sprague-Dawley	M/(300–400) g	NA	Full thiness, a 100 °C brass comb for 30s	R-BMSCs	1 × 10^6^ cells/wound	Tail vein	60 m
Liu	2014	China	84	42	42	Rat/Wistar	M/(180–220) g	6 W	Full thiness (30% TBSA), 94 °C water for 12 s	H-UCMSCs	5 × 10^6^ cells/wound	Tail vein	72 h
Zhang	2015	China	84	42	42	Rat/Sprague-Dawley	M/(200 ± 5) g	NA	Full thiness, burned iron for 25 s	H-UCMSCs	2 × 10^6^ cells/animal	Local injection	24 h
Clover*	2015	Ireland	3	36	36	Pig/Landrace	F/(25–30) kg	NA	Partial thickness (5% TBSA), a 80 °C brass block for 30 s	Allogenic P-BMSCs	4.5 × 10^6^ cells/wound	Cover	Immediately
Abbas	2018	Turkey	50	20	20	Rat/Wistar	M/(300–325) g	Adult	Thickness unavailable, a 100 °C brass comb for 30 s	R-BMSCs	1 × 10^6^ cells/wound	Local injection	30 m
Alapure	2018	New Orleans	8	4	4	Mice/C57BL/6 J	F/NA	9 W	Full thiness, a 100 °C alloy-aluminum-rod for 4 s	M-BMSCs seeded AC gels	1 × 10^5^ cells/wound	Local injection	48 h
Banerjee	2019	USA	8	4	4	Rat/Harlan	M/NA	8–10 W	Partial thickness, a 87 °C brass for 10s	R-ADSCs	5 × 10^4^ cells/wound	Local injection	9d
Chang*	2018	Taiwan	20	28	12	Rat/Wistar	M/(176–250) g	6–7 W	Full thiness (20% TBSA), a 95 °C metal rod	R-ADSCs	5 × 10^6^ cells/wound	Local injection	24 h
Feng*	2019	Taiwan	6	9	9	Rat/Sprague-Dawley	NA/(250–300) g	NA	Partial thickness, a 90 °C copper plate for 30 s	R-ADSCs	5 × 10^5^ cells/wound	Local injection	30 m
Foubert*	2018	USA	15	16	14	Pig/Yorkshire swine	F/(80–130) g	4-6 M	Full thickness/Partial thickness (<10% TBSA), a 180–200 °C brass template for 30 s/60 s	Autologous P-ADSCs	0.5 × 10^6^ cells/kg	Local injection	5 m
Kaita	2019	Japan	24	12	12	Mice/BALB/c Nude	M/NA	6–8 W	Full thickness, a 150 °C for 5 s	H-ADSCs	5 × 10^4^ cells/wound	Local injection	1 h
Mahmood*	2018	Pakistan	12	12	12	Rat/Sprague-Dawley	NA/NA	9–12 M	Full thickness, a 100 °C brass rod (2x2cm) for 20s	H-UCMSCs	3 × 10^4^ cells/wound	Cover	48 h
Oryan*	2019	Iran	8	16	16	Rat/Sprague-Dawley	M/(200 ± 20)g	7–9 W	Full thickness, a 100 °C aluminum bar for 10 s	Allogenic R-ADSCs	1 × 10^6^ cells/wound	Local injection	48 h
Yang	2018	China	40	30	10	Mice/BALB/c	M/F/NA	Adult	Full thickness, a 100 °C copper plate tip for 10 s	H-UCMSCs	8 × 10^5^ cells/wound	Local injection	5 m
Liao	2019	China	72	24	24	Rat/Sprague-Dawley	M/NA	8 W	Partial thickness, 100 °C water	M-ADSCs	2 × 10^6^ cells/wound	Cover	NA

*USA* United States of America, *MSC* mesenchymal stem cells, *Con* control, *AD* administration, *TBSA* total body surface area, *H-BMSCs* human bone marrow stem cells, *M-ADSCs* mouse adipose-derived mesenchymal stem cells, *H-ADSCs* human adipose-derived mesenchymal stem cells, *R-BMSCs* rat bone marrow stem cells, *H-UCMSCs* human umbilical cord mesenchymal stem cells, *P-BMSCs* pig bone marrow stem cells, *M-BMSCs* mouse bone marrow stem cells, *R-ADSCs* rat adipose-derived mesenchymal stem cells, *P-ADSCs* pig adipose-derived mesenchymal stem cells, *W* week, *M* month, *M* male, *F* female, *NA* not available, *m* minute, *h* hour, *d* day

*Means there are more than one burn wounds in a rat, mouse, or pig

### Primary results

#### Closure rate

Our primary goal was to assess whether stem cells had a therapeutic effect on burn wound healing, while the primary outcome was composed of three aspects: closure rate, un-healing rate of the wound area, and wound area. The closure rate and un-healing rate of the wound area were expressed as percentages of the area, and the wound area was expressed as the actual area of the burned site.

**Fig. 2:**
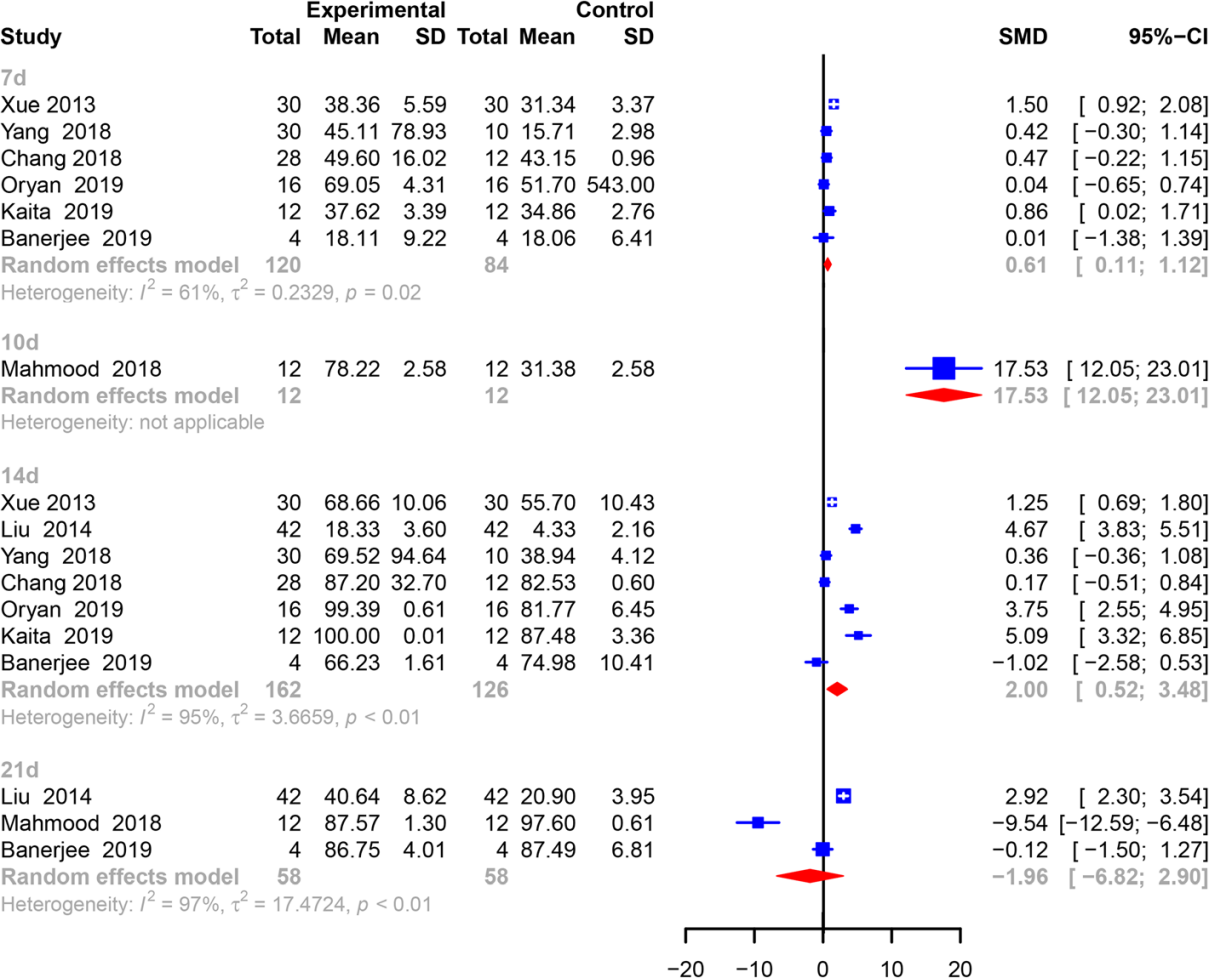
Primary outcome of closure rate. The outcome of the closure rate was analyzed at four different time points. All analyses showed positive results for the shrinking closure rate (*p* < 0.05). All analyses were conducted by using a random-effects model with a 95% confidence interval

Closure rates were reported by 8 studies. We divided the outcome of closure rate into 4 groups: 7 days (*n* = 6), 10 days (*n* = 1), 14 days (*n* = 7), and 21 days (*n* = 3). In total, 256 animals and 312 burn wounds were included in the analysis (Fig. 2). Only Mahmood [10] reported the closure rate on the 10th day; thus, the result from Mahmood was not pooled. On the 7th day, animals in the MSC group already had a higher closure rate (0.61, 95% CI 0.11 to 1.12, *p* = 0.002) than animals in the control group. On the 14th day, the outcome shared similar significance (2.00, 95% CI 0.52 to 3.48, *p* = 0.008) with the outcome on the 7th day. However, no significant difference (− 1.96, 95% CI − 6.82 to 2.90, *p* = 0.428) in the closure rate was observed between the two groups on the 21st day.

A leave-one-out meta-analysis was also conducted to determine that elimination of articles by Kaita [25] changed the outcome of the closure rate to nonsignificance (0.56, 95% CI − 0.04 to 1.16) on the 7th day (see Additional file 3). On the 14th day, no article was observed to greatly impact the final outcome of closure rate (see Additional file 4).

#### Wound area

**Fig. 3:**
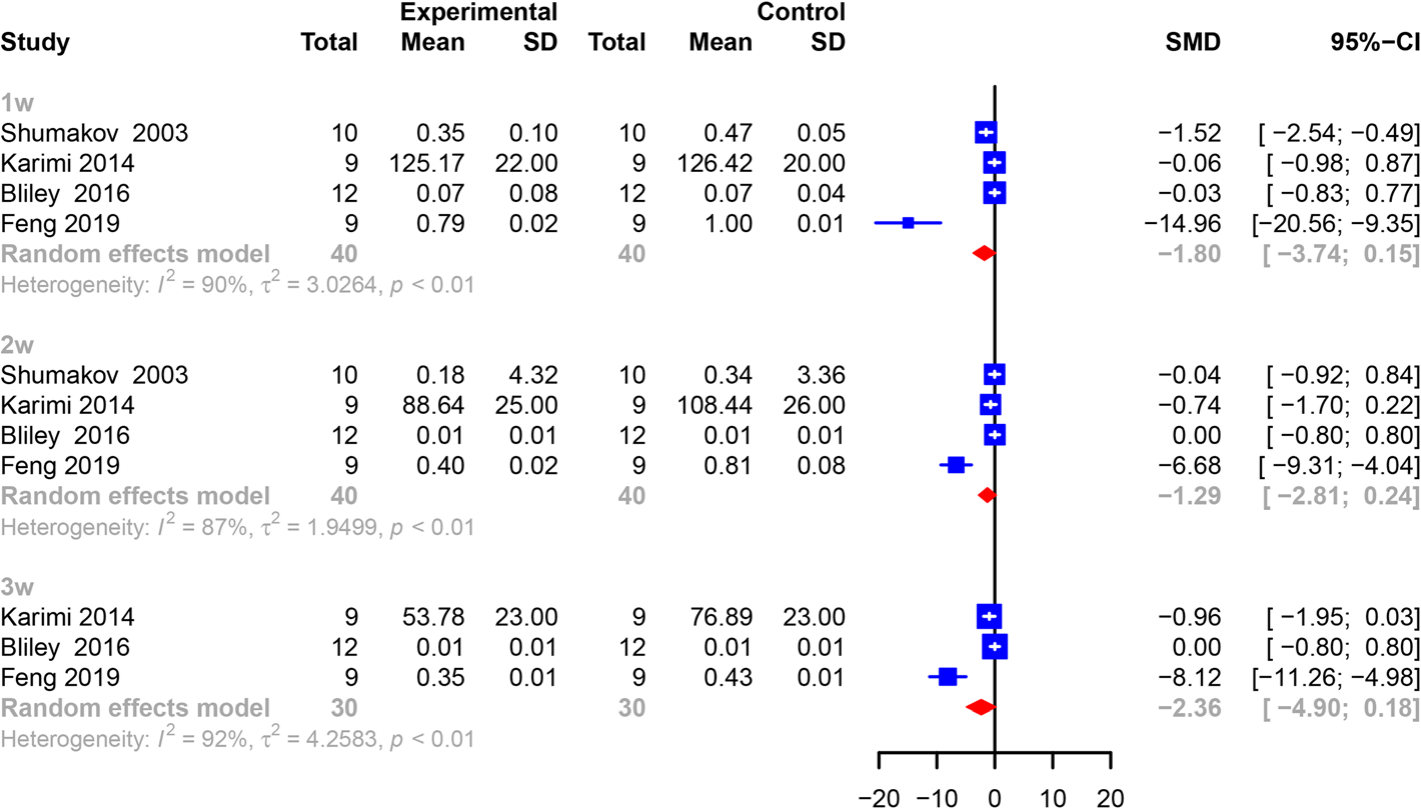
Primary outcome of the wound area. The outcome of closure rate was analyzed every other week, and a negative association was observed between MSC administration and wound area. All analyses were conducted by using a random-effects model with a 95% confidence interval

Four studies, including 62 animals and 80 burn wounds, reported the outcome of wound area at the 1st and 2nd week. This indicator was no longer reported by Shumakov [26] but was reported by three other authors at the 3rd week. Similarly, we observed that wound area was negatively associated with stem cell therapy at the 1st (− 1.80, 95% CI − 3.74 to 0.15, *p* = 0.070), 2nd (− 1.29, 95% CI − 2.81 to 0.24, *p* = 0.098), and 3rd weeks (− 2.36, 95% CI − 4.90 to 0.18, *p* = 0.069). Although nonsignificance was discovered at each time point, we observed that the *p* value from each group approached 0.05, which suggested a closing trend of the wound area (Fig. 3).

A leave-one-out meta-analysis revealed that the wound area greatly decreased if articles by Karimi (− 3.30, 95% CI − 6.40 to − 0.19) or Bliley (− 3.40, 955 CI − 6.61 to − 0.19) were omitted at the 1st week (see Additional file 5). However, at the 2nd and 3rd weeks, no article that significantly impacted the final outcome was observed (see Additional files 6 and 7).

#### Un-healing rate of wound area

**Fig. 4:**
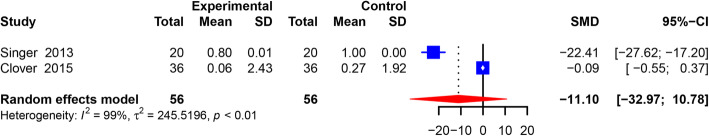
Primary outcome of the un-healing rate of the wound area. The pooled analysis suggested that both groups shared a similar closure rate of un-healing of the wound area. The analysis was conducted by using a random-effects model with a 95% confidence interval

The un-healing rate of the wound area was only reported by 2 articles, including 23 animals and 112 burn wounds. Animals in the experimental group had the same un-healing rate of the wound area as the animals in the control group (95% CI − 32.97 to 10.78, *p* = 0.320) (Fig. 4).

### Secondary results

In this section, we primarily focused on biochemical events (such as the white blood cell count, eschar thickness, CD-31, vascular density, IL-10, and VEGF) that occurred following stem cell administration (Table 2). A total of 5 comparative studies involving 127 animals and 178 burn wounds evaluated the effect of MSCs on the thickness of the eschar tissue and the vascular density. The pooled analysis showed that experimental animals had a thicker scar (6.56, 95% CI 1.115 to 11.98, *p* = 0.017) and a higher vascular density (4.69, 95% CI 0.06 to 9.31, *p* = 0.047)) than animals administered no treatment and animals treated with phosphate buffer solution (PBS). Interestingly, we observed a negative result in CD-31 staining (10.02, 95% CI − 10.33 to 30.37, *p* = 0.335), which was contradictory to the pooled analysis of vascular density.

**Table 2:** Secondary outcomes

Item	SMD	95% CI	*P* value
Thickness of eschar	6.56	[1.15;11.98]	***0.017***
CD-31	10.02	[− 10.33;30.37]	0.335
Vascular density	4.69	[0.06;9.31]	***0.047***
IL-10	9.82	[−23.78;43.41]	0.567
VEGF	−2.95	[−9.55;3.65]	0.381

*SMD* standardized mean difference, *IL* interleukin, *CD-31* cluster of differentiation-31, *VEGF* vascular endothelial growth factor

**Fig. 5:**
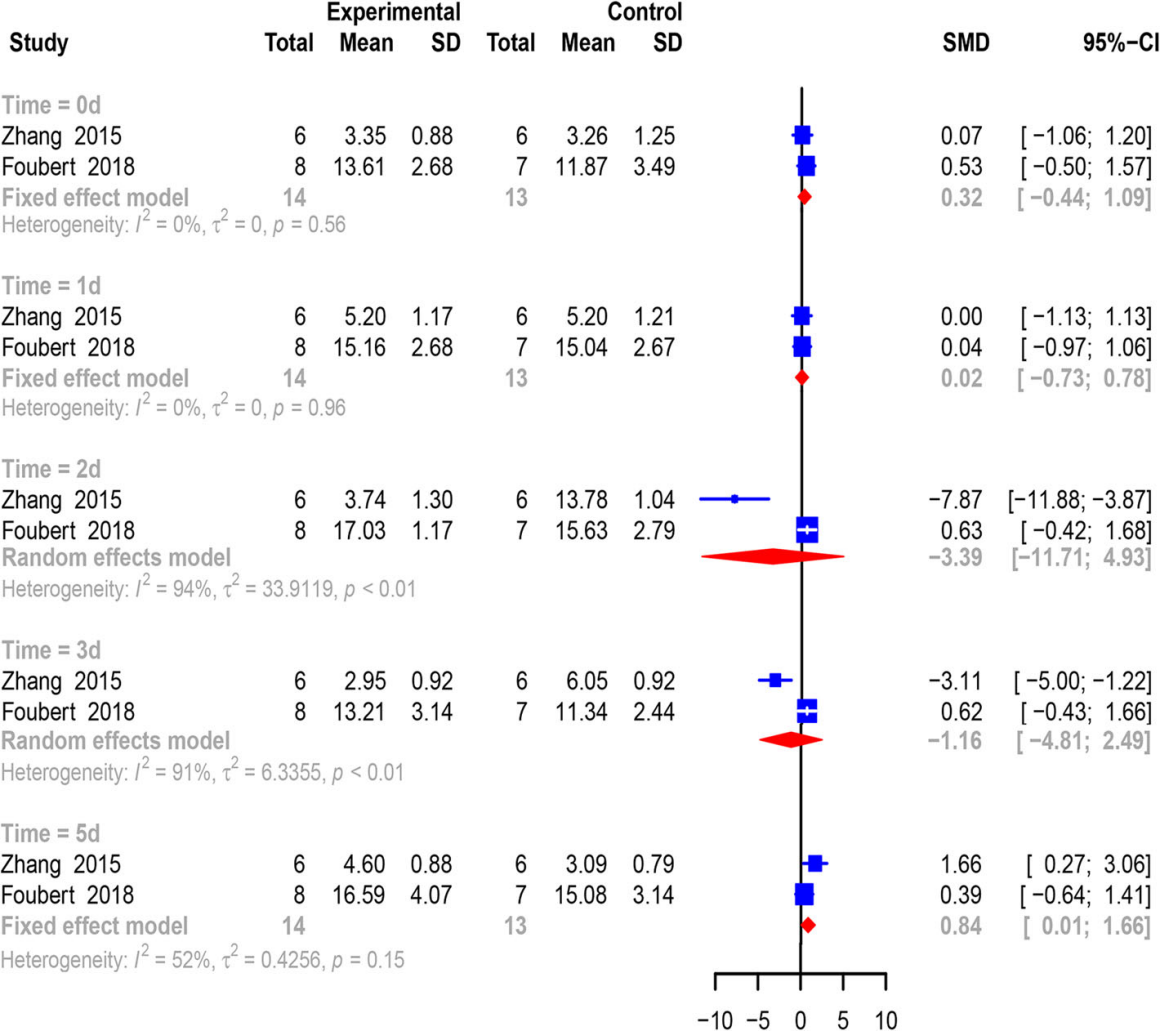
Pooled analysis of the white blood cell count. Significant changes in white blood cells were not observed within 3 days. On the fifth day, white blood cells were greatly elevated in animals receiving MSC administration. All analyses were conducted by using a random- or fixed-effects model with a 95% confidence interval

Both Zhang and Foubert [27, 28] continuously reported changes in the white blood cell count of 27 animals at 0, 1, 2, 3, and 5 days post treatment (Fig. 5). At the 0 (0.32, 95% CI − 0.44 to 1.09, *p* = 0.409), 1st (0.02, 95% CI − 0.73 to 0.78, *p* = 0.953), 2nd (− 3.39, 95% CI − 11.71 to 4.93, *p* = 0.424), and 3rd day (− 1.16, 95% CI − 4.81 to 2.49, *p* = 0.533) after treatment, no significant change in the white blood cell count was discovered in the MSC group until the 5th day post treatment. On the last day, an elevated count of white blood cells (0.84, 95% CI 0.01 to 1.66, *p* = 0.047) was observed in animals administered MSCs.

Other factors, such as IL-10 (9.82, 95% CI − 23.78 to 43.41, *p* = 0.567) and VEGF (− 2.95, 95% CI − 9.55 to 3.65, *p* = 0.381), were not significantly increased in animals in the MSC group.

### Subgroup analysis

To identify heterogeneity potentially influencing the analysis of the healing process, articles were divided into several groups on the basis of year, species, cell type, cell origin, gender, and region. On the 7th day, pooled analysis of articles within 5 years showed a 0.39 higher closure rate (95% CI 0.03 to 0.74, *p* = 0.000) in the MSC group. MSC therapy seemed to significantly elevate the closure rate for mice regardless of MSC type (1.03, 95% CI 0.63 to 1.43, *p* = 0.000) and animals treated with MSCs of human origin (0.65, 95% CI 0.04 to 1.26, *p* = 0.038). Stem cells from rats did not show a curative effect on the closure rate (0.23, 95% CI − 0.23 to 0.69, *p* = 0.325). In articles published by Asian authors (0.67, 95% CI 0.13 to 1.21, *p* = 0.015) and in studies involving male animals (0.65, 95% CI 0.04 to 1.26, *p* = 0.038), there was also a significant difference in closure rate between the two groups (Table 3) at 14 days post treatment (DPT). Even though a pooled analysis resulted in no significant differences between the two groups regardless of whether the articles were from within 5 years (*p* = 0.070) or beyond 5 years (*p* = 0.085), a trend of significance was discovered. Moreover, for mice treated with all sorts of MSCs (3.33, 95% CI 0.01 to 6.65, *p* = 0.049) and animals treated with MSCs of human origin (3.33, 95% CI 0.01 to 6.65, *p* = 0.049), a significant difference in the closure rate between the two groups was identified. On the 14th day, Asian articles also showed a great improvement in closure rate (2.46, 95% CI 0.89, 4.02, *p* = 0.002) (Table 4).

**Table 3:** Subgroup analysis for the closure rate at 7 DPT

Subgroup	SMD	95% CI	***P*** value
**Year**			
Within 5 years	0.39	[0.03,0.74]	***0.032***
**Species**			
Mouse	1.03	[0.63,1.43]	***0.000***
Rat	0.23	[−0.23,0.69]	0.325
**Cell type**			
ADSCs	0.23	[− 0.23,0.69]	0.325
**Cell origin**			
Human	1.03	[0.63,1.43]	***0.000***
Rat	0.23	[−0.23,0.69]	0.325
**Gender**			
Male	0.65	[0.04,1.26]	***0.038***
**Region**			
Asia	0.67	[0.13,1.21]	***0.015***

*DPT* days post treatment, *SMD* standardized mean difference

**Table 4:** Subgroup analysis for the closure rate at 14 DPT

Subgroup	SMD	95% CI	***P*** value
**Year**			
Beyond 5 years	2.95	[−0.41,6.03]	***0.085***
Within 5 years	1.59	[−0.13,3.32]	***0.070***
**Species**			
Mouse	3.33	[0.01,6.65]	***0.049***
Rat	1.07	[−0.39,2.52]	0.151
**Cell type**			
ADSCs	1.07	[−0.39,2.52]	0.151
**Cell origin**			
Human	3.33	[0.01,6.65]	***0.049***
Rat	1.07	[−0.39,2.52]	0.151
**Gender**			
Male	2.29	[0.55,4.03]	***0.010***
**Region**			
Asia	2.46	[0.89,4.02]	***0.002***

*DPT* days post treatment, *SMD* standardized mean difference

For the wound area, subgroup analysis of species, cell origin, and cell type at the 1st, 2nd, and 3rd week was conducted. Regardless of the period post treatment, no factor of species, cell origin, and cell type had a significant impact on the final outcome of the wound area. However, the wound area tended to decrease in animals treated with adipose-derived stem cells at the 1st (− 2.42, 95% CI − 5.22 to 0.38, *p* = 0.091) and 2nd (− 2.01, 95% CI − 4.34 to 0.32, *p* = 0.091) weeks (Table 5).

**Table 5:** Subgroup analysis for the wound area at 1st, 2nd, and 3rd weeks post treatment

Subgroup	SMD	95% CI	***P*** value
**1st**			
**Species**			
Mouse	−0.04	[−0.65,0.56]	0.8895
Rat	−7.94	[−21.10,5.22]	0.2369
**Cell origin**			
Mouse	−7.24	[−21.83,7.35]	0.3309
**Cell type**			
ADSCs	−2.42	[−5.22,0.38]	***0.0908***
**2nd**			
**Species**			
Mouse	−0.32	[−1.04,0.40]	0.3837
Rat	−3.24	[−9.74,3.26]	0.3289
**Cell origin**			
Mouse	−3.58	[−9.39,2.24]	0.2279
**Cell type**			
ADSCs	−2.01	[−4.34,0.32]	***0.0912***
**3rd**			
**Species**			
Mouse	−0.43	[−1.37,0.50]	0.3639
**Cell origin**			
Mouse	−4.38	[−11.39,2.63]	0.2212

*SMD* standardized mean difference

### Publication bias

**Fig. 6:**
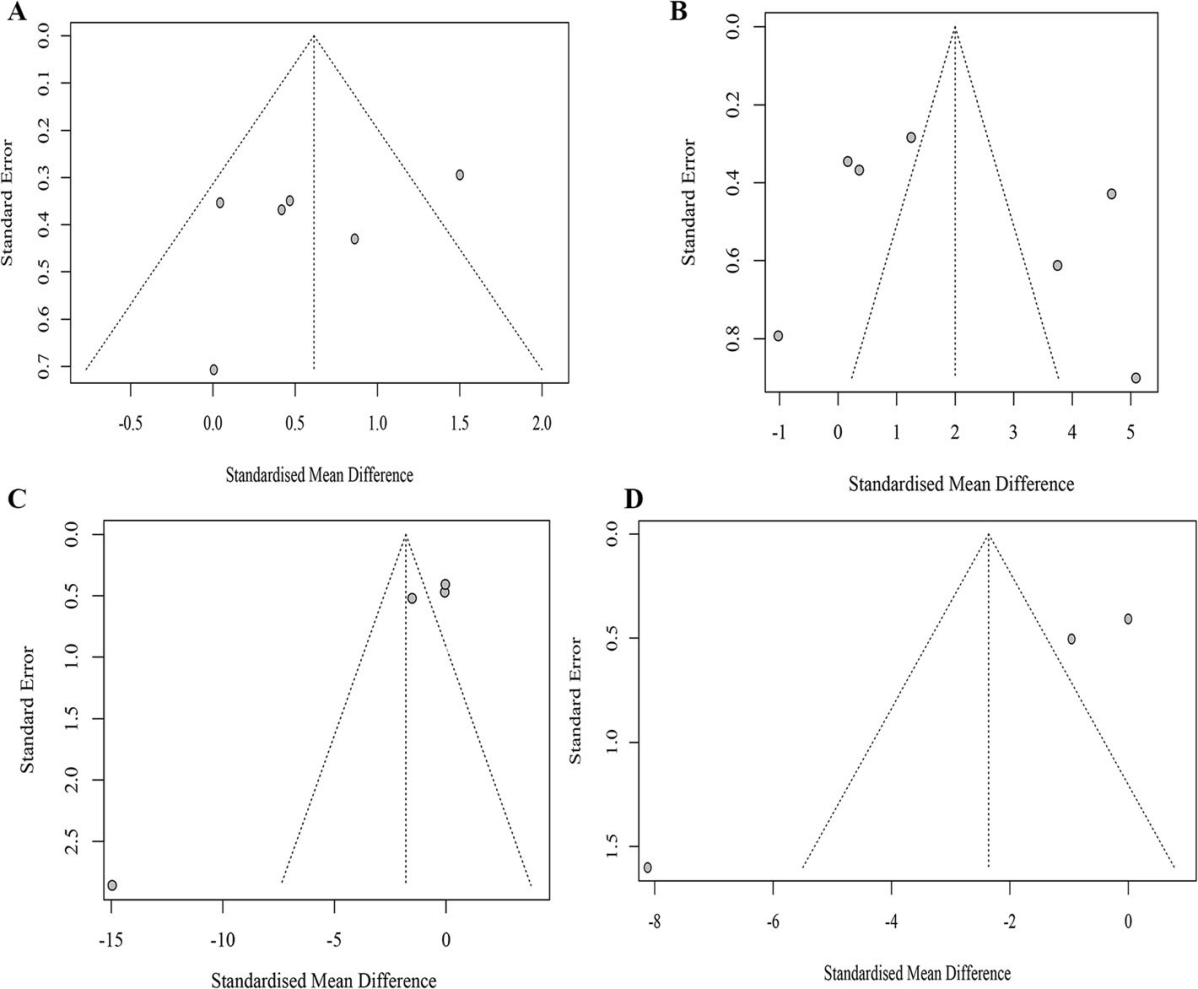
Funnel plots of primary outcomes. Funnel plots were conducted for closure rate on the 7th (**a**) and 14th (**b**) days and for the wound area on the 1st (**c**) and 3rd (**d**) weeks

Publication bias was tested by funnel plots (Fig. 6) and Egger’s linear regression in closure rate and wound area individually. As the number of included studies that measured the closure rate and wound area was small (< 10), it remained unclear whether the scattered points were the result of publication bias or the limited number of articles.

No significant publication bias was observed for the closure rate on the 7th (Egger’s test, *p* = 0.329) or 14th (Egger’s test, *p* = 0.4316) days. Although significant publication bias was discovered for wound area at the 3rd week (Egger’s test, *p* = 0.060) post treatment, significant publication bias was observed for wound area at the 1st week (Egger’s test, *p* = 0.045) post treatment, which may be the result of the small sample effect.

Furthermore, the outcomes of meta-regression demonstrated that species (*p* = 0.058), cell type (*p* = 0.005), cell origin (*p* = 0.058), and year (*p* = 0.001) were potential heterogeneous factors, rather than gender (*p* = 0.757) or region (*p* = 0.463), for closure rate on the 7th day. However, on the 14th day, year (*p* = 0.708), gender (*p* = 0.402), species (*p* = 0.163), cell type (*p* = 0.185), cell origin (*p* = 0.163), and region (*p* = 0.114) were not potential heterogeneous origins. For wound area, both cell origin (*p* = 0.7324) and cell type (*p* = 0.8212) were not potential heterogeneous factors, in contrast with species (*p* = 0.028), at the 1st week. However, cell origin (*p* = 0.692), cell type (*p* = 0.390), and species (*p* = 0.236) were not heterogeneous factors for wound area at the 2nd week. At the 3rd week, the meta-regression analysis suggested that species (*p* = 0.00), rather than cell origin (*p* = 0.473), was the source of potential heterogeneity.

## Discussion

When combined with a meta-analysis of these experiments, a systematic review enables a more systematic and objective assessment of the results. Severe burns are one of the most devastating injuries [29, 30]. Burns are one of the most common traumas worldwide, with more than 300,000 people dying each year from fire-related burns and millions suffering from burn-induced deformities and mortality [31].

Stem cells are self-renewing cells that can differentiate into specific cell types. Pluripotent stem cells, i.e., ESC or induced pluripotent stem cells (iPSCs), differentiate into all three embryonic lines of cells. Since the 1970s, MSCs have been called mesenchymal stromal cells, and numerous achievements have been made in the field of regenerative therapy using these cells [32]. MSCs have some important advantages over other types of stem cells. MSCs are clongenic and able to differentiate into various cell lineages, including muscle, cartilage, fat, and bone [33], and they are multipotent, releasing a wide range of biologically active paracrine factors. Furthermore, MSCs have documented immunomodulatory properties and can be isolated with relative ease from a variety of adult tissues, such as bone marrow, tendon, adipose tissue, skin, and periodontal ligament [34–39]. Importantly, this flexibility in where MSCs can be harvested avoids ethical issues associated with the use of stem cells from embryonic cells. Finally, the potential risk of stem cell therapy is their ability to become malignant, which is especially true for iPSCs. In contrast, this unique risk appears to be reduced in MSC therapy. Previous studies have suggested that MSCs can significantly attenuate inflammation [40, 41] and tissue fibrosis, promote tissue remodeling despite the use of bleomycin treatment [42], and shorten the healing process with less scar formation in burn patients [43]. Over the years, the use of MSCs to accelerate burn wound closure has shown promising prospects [44–47]. The potential mechanisms associated with burn treatment could be attributed to abundant anti-inflammatory cytokines secreted by MSCs, such as VEGF, EGF, FGF, IGF, PDGF, TGF, IL-4, IL-6, and IL-10. Moreover, MSC treatment significantly reduces neutrophil infiltration and the levels of pro-inflammatory cytokines (TNFα, IL-1β, and IL-6) while promoting anti-inflammatory cytokine IL-10 expression in the stagnation region [48]. Additionally, MSC-treated interstitial spaces exhibit increased expression of VEGF-A, PDGF, FGF, and TGF, increasing the vascular density in the lesion site [49].

Nevertheless, to the best of our knowledge, this is the first attempt to comprehensively collect and evaluate the current preclinical evidence supporting the use of MSCs in animal models of burn wounds. Our results demonstrated that MSCs are potentially therapeutic in burn animal models.

Overall, the results of our meta-analysis revealed an improvement in the wound closure rate and a trend toward shrinkage in the wound area. Pooled analysis of only two articles suggested a negative result in the un-healing rate of the wound area, which was an aspect that measured the degree of the burn injuries and led to a controversial conclusion. However, two articles may not firmly support this controversial conclusion, which was even inconsistent with the results reported by Motamed et al. [50] Sensitivity analysis showed a more stable outcome on the 14th day than the outcome of closure rate on the 7th day, as the omission of a study by Kaita [25] significantly impacted the result. We also evaluated the eschar thickness and vascular density in animals treated with regenerative cells, which were obviously better and consistent with the research results of Sultan et al. [51] Interestingly, animals receiving MSCs did not exhibit increased expression of CD-31, which may be explained by limited articles. Taken together, these findings support the potential use of MSC therapy in preclinical applications of burn wounds. To explore sources of heterogeneity, subgroup analyses and meta-regression were conducted on the basis of species, cell origin, cell type, gender, region, and year. The analysis revealed that these variables were exceedingly relevant for future patient application, species, cell type, and cell origin and were potential sources of heterogeneity; thus, the process of generalizing MSC therapy in humans may be conducted more cautiously. These findings provide a certain reference significance, which reminds us of some species- and cell-related issues that are pertinent to future preclinical or clinical trials, and these variables need to be taken into account to determine the best successful outcome.

Despite the exclusion of other cell-based studies, we reviewed the efficacy of other cell-based therapies in the treatment of burn wounds. Shi [52] observed that the healing rate of the ESC group on days 7 and 14 was 26.0 ± 2.0 and 64.4 ± 4.7%, respectively, which was significantly higher than that of the control group (12.4 ± 1.1 and 29.1 ± 3.3%). Khan [53] also observed that the treatment of wounds with iPSCs significantly promoted the rate of wound healing closure compared to that observed in other groups (*p* < 0.05). The mechanism by which HSCs accelerate the wound healing process potentially involves the secretion of collagen and downregulation of matrix metalloproteinase (MMP) expression [54]; meanwhile, endothelial progenitor cells (EPCs) participate in this process by promoting vessel density [55]. While all of the abovementioned cells can accelerate wound healing, the cell type that is the most suitable for burn wound healing remains unclear.

Despite positive findings in small animal models, the conversion to large animal models and clinical research has been limited. Of the three case human reports, no study described adverse effects. In a study by Jeschke et al [20], a male patient in his mid-20s with a 70% TBSA burn injury had wounds that remained unhealed after more than 18 months of routine burn care. With the administration of allogeneic MSCs, the open wound was reduced from one third to less than 3%, and the infection was significantly cured in a short time. Furthermore, the wound sites showed no evidence of keloids or hypertrophic formation during a 6-year follow-up period. Another clinical trial by Wael et al. [23] also observed a significantly reduced hospitalization period in both the BM-MSCs and UC-MSCS groups as compared to the EE&G group, indicating that mesenchymal stem cells of both bone marrow and cord blood origin can effectively improve the healing of burn injuries. Furthermore, all these studies described rapid improvement in overall clinical condition. However, the number of available studies is too small to enable a meta-analysis of the clinical application of MSC. One phase I clinical trial (NCT02104713) is currently underway in the USA to determine the safety of allogeneic stem cell therapy from healthy donors for 2nd degree burn wounds of less than 20% TBSA at four different dose levels (ranging from 2.5 × 10^3^ to 2 × 10^4^ allogeneic BM-MSCs/cm^2^) [56].

In addition to being used to treat burns, MSCs and other cells are also used to treat other traumatic and regenerative diseases alone or in combination with other agents (i.e., platelet-rich plasma (PRP) and hyaluronic acid). Hair follicle mesenchymal stem cells (HF-MSCs), human intra- and extradermal adipose tissue-derived hair follicle stem cells (HD-AFSCs), and stem cells with PDGF present safe and viable treatment alternatives against hair loss [57, 58]. In addition, PRP alone or in combination with hyaluronic acid can be used to regenerate lower-extremity complex wounds, treat severe hidradenitis suppurativa, and protect against hair loss [59–61]. Additionally, PRP is effective in the treatment of jaw regeneration, the protection of HUVECs, and promotion of the chondro/osteogenic differentiation of ADMSCs in the presence of insulin [62–64]. Gentile et al. observed that transplantation of nanofat was positively correlated with scar regeneration, and even the transplantation of Permacol, a porcine dermal matrix, was effective in treating an infected abdomen wound [65, 66]. The regenerative effects of these cells could be attributed to paracrine through the secretion of various growth factors and the exocytosis of exosomes. Growth factors, such as EGF, FGF, and VEGF, strongly activate their receptor pathways to induce proliferation-associated pathways (Akt and Erk) and promote cell proliferation [67]. Furthermore, these cytokines (i.e., exosomes) can distinctly suppress inflammation cascades in the wound site, blocking the expansion of the secondary injury [68].

The advantages of this investigation are apparent. First, we are the first to conduct a meta-analysis of the impact of MSC therapy on burn wounds. Second, we conducted a systematic literature search and followed the published protocols to ensure a stringent review process. Third, the detailed analysis adds variables that the researcher needs to pay attention to.

Although our meta-analysis showed that MSC transplantation was effective in animal burn wound healing, some limitations should be taken into consideration. First, the results are from various species of animals rather than humans, which may lead to poor reproducibility from preclinical to clinical trials. Furthermore, some subgroups had a small sample size. Additionally, the overall number of studies is small. Additionally, we could not assess the quality of studies such as those investigating humans. Finally, we cannot evaluate the clinical safety of MSCs in treating burn wounds owing to the lack of reported side-effect events and the studied objectives. Finally, there was no in-depth study on the dose effect and administration method of MSCs on burn wounds.

## Conclusion

Our pooled analysis showed that MSC therapy, which is an emerging treatment for burn wounds, is therapeutic in accelerating the process of wound healing in animals, and this study provides some insights into the clinical application of stem cell therapy. Owing to a lack of assessment of side-effect events for MSCs, safety issues should receive greater focus while clinical trials are ongoing.

## Supplementary information

**Additional file 1.** PRISMA 2009 Checklist.

**Additional file 2.** Search Strategy.

**Additional file 3.** Leave-one-out meta-analysis of the closure rate on the 7th day. We conducted a sensitivity analysis by using a leave-one-out meta-analysis, showing that no single article (except for one study conducted by Kaita) significantly impacted the final pooled outcome of closure rate.

**Additional file 4.** Leave-one-out meta-analysis of the closure rate on the 14th day. The leave-one-out meta-analysis calculated with random-effects models showed that the final closure rate was so stable that it could be significantly impacted by a single study.

**Additional file 5.** Leave-one-out meta-analysis of the wound area at the 1st week. The leave-one-out meta-analysis calculated with random-effects models showed that the final result of the wound area could be easily affected by two studies conducted by Karimi and Bliley.

**Additional file 6.** Leave-one-out meta-analysis of the wound area at the 2nd week. Leave-one-out meta-analysis calculated with random-effects models showed that the pooled outcome of the wound area was not significantly changed by a single article.

**Additional file 7.** Leave-one-out meta-analysis of the wound area at the 3rd week. Leave-one-out meta-analysis calculated with random-effects models showed that the pooled outcome of the wound area was not significantly changed by a single article.

## Data Availability

Not applicable.

## Abbreviations

USA: United States of America
MSC: Mesenchymal stem cells
Con: Control
AD: Administration
TBSA: Total body surface area
H-BMSCs: Human bone marrow stem cells
M-ADSCs: Mouse adipose-derived mesenchymal stem cells
H-ADSCs: Human adipose-derived mesenchymal stem cells
R-BMSCs: Rat bone marrow stem cells
H-UCMSCs: Human umbilical cord mesenchymal stem cells
P-BMSCs: Pig bone marrow stem cells
M-BMSCs: Mouse bone marrow stem cells
R-ADSCs: Rat adipose-derived mesenchymal stem cells
P-ADSCs: Pig adipose-derived mesenchymal stem cells
W: Week
M: Month
M: Male
F: Female
NA: Not available
m: Minute
h: Hour
d: Day
WBCs: White blood cells
SD: Standard difference
SMD: Standardized mean difference
IL: Interleukin
CD-31: Cluster of differentiation-31
VEGF: Vascular endothelial growth factor
DPT: Days post treatment
CI: Confidence interval
WHO: World Health Organization
EGF: Epidermal growth factor
FGF: Fibroblast growth factor
IGF: Insulin-like growth factor
PDGF: Platelet-derived factor growth factor
TGF beta: Transforming growth factor beta
PRISMA: Preferred Reporting Items for Systematic Review and Meta-Analysis
ESCs: Embryonic stem cells
RCT: Randomized clinical trials
AOD: Average optical density
RT-QPCR: Reverse transcription-polymerase chain reaction
ELISA: Enzyme-linked immunosorbent assay
PBS: Phosphate buffer solution
iPSCs: Induced pluripotent stem cells
MMP: Matrix metalloproteinase
EPCs: Endothelial progenitor cells
PRP: Platelet-rich plasma
HF-MSCs: Hair follicle mesenchymal stem cells
HD-AFSCs: Human intra- and extradermal adipose tissue-derived hair follicle stem cells
