# Supplementary material for: Efficacy assessment of mesenchymal stem cell transplantation for burn wounds in animals: a systematic review
Source: Stem Cell Res Ther. 2020 Aug 28;11:372. doi: 10.1186/s13287-020-01879-1 (PMC7456061; doi:10.1186/s13287-020-01879-1)
Supplement: Supplementary file 2 — Additional file 2. Search Strategy. [file 13287_2020_1879_MOESM2_ESM.docx]

**Additional file 2. Search Strategy**

| **Electronic databases** | **Search** | **Search strategy** | **Results** |
| --- | --- | --- | --- |
| **PubMed** | #1 | (mesenchymal stem cells [Title/Abstract]) OR (MSCs [Title/Abstract]) | 45981 |
|  | #2 | burn | 36952 |
|  | #3 | #1 AND #2 | **347** |
| **Medline** | #1 | (mesenchymal stem cells [Title/Abstract]) OR (MSCs [Title/Abstract]) | [111959](https://www.ncbi.nlm.nih.gov/pubmed/?cmd=HistorySearch&querykey=5) |
|  | #2 | burn | [377045](https://www.ncbi.nlm.nih.gov/pubmed/?cmd=HistorySearch&querykey=6) |
|  | #3 | #1 AND #2 | [**126**](https://www.ncbi.nlm.nih.gov/pubmed/?cmd=HistorySearch&querykey=11) |
| **EMBASE** | #1 | 'mesenchymal stem cells’: ti,ab,kw OR mscs:ti,ab,kw | 52482 |
|  | #2 | burn:ti,ab,kw | 39377 |
|  | #3 | #1 AND #2 | **93** |
| **Cochrane Library** | #1 | (mesenchymal stem cells):ti,ab,kw OR (MSCs):ti,ab,kw | 4 |
|  | #2 | burn:ti,ab,kw | 127 |
|  | #3 | #1 AND #2 | **15** |
| **Web of Science** | #1 | TS= (mesenchymal stem cells) OR TS=(MSCs) | 72038 |
|  | #2 | TS=(burn) | 10421 |
|  | #3 | #1 AND #2 | **104** |
