# Supplementary figures and images for: Efficacy assessment of mesenchymal stem cell transplantation for burn wounds in animals: a systematic review
Source: Stem Cell Res Ther. 2020 Aug 28;11:372. doi: 10.1186/s13287-020-01879-1 (PMC7456061; doi:10.1186/s13287-020-01879-1)

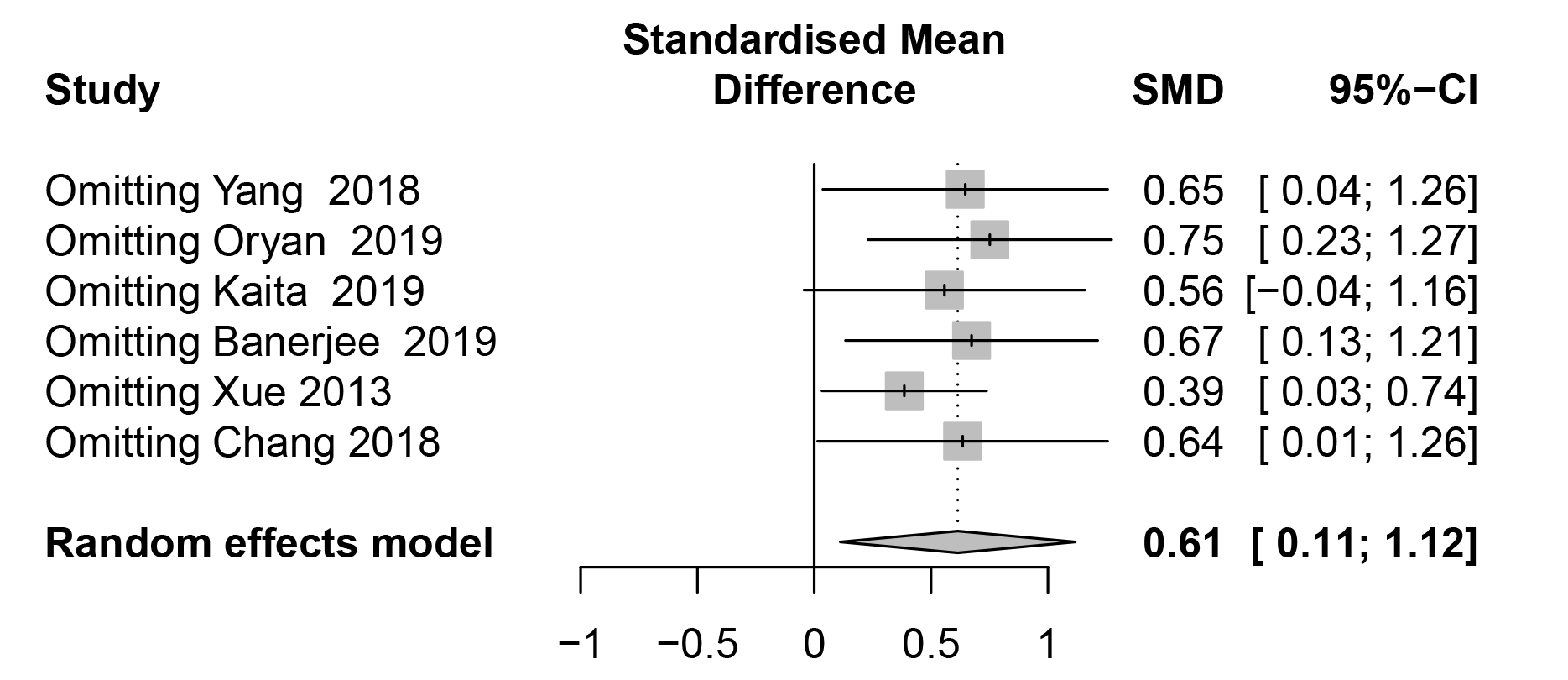

Supplement: Supplementary file 3 — Additional file 3. Leave-one-out meta-analysis of the closure rate on the 7th day. We conducted a sensitivity analysis by using a leave-one-out meta-analysis, showing that no single article (except for one study conducted by Kaita) significantly impacted the final pooled outcome of closure rate. [file 13287_2020_1879_MOESM3_ESM.tif]

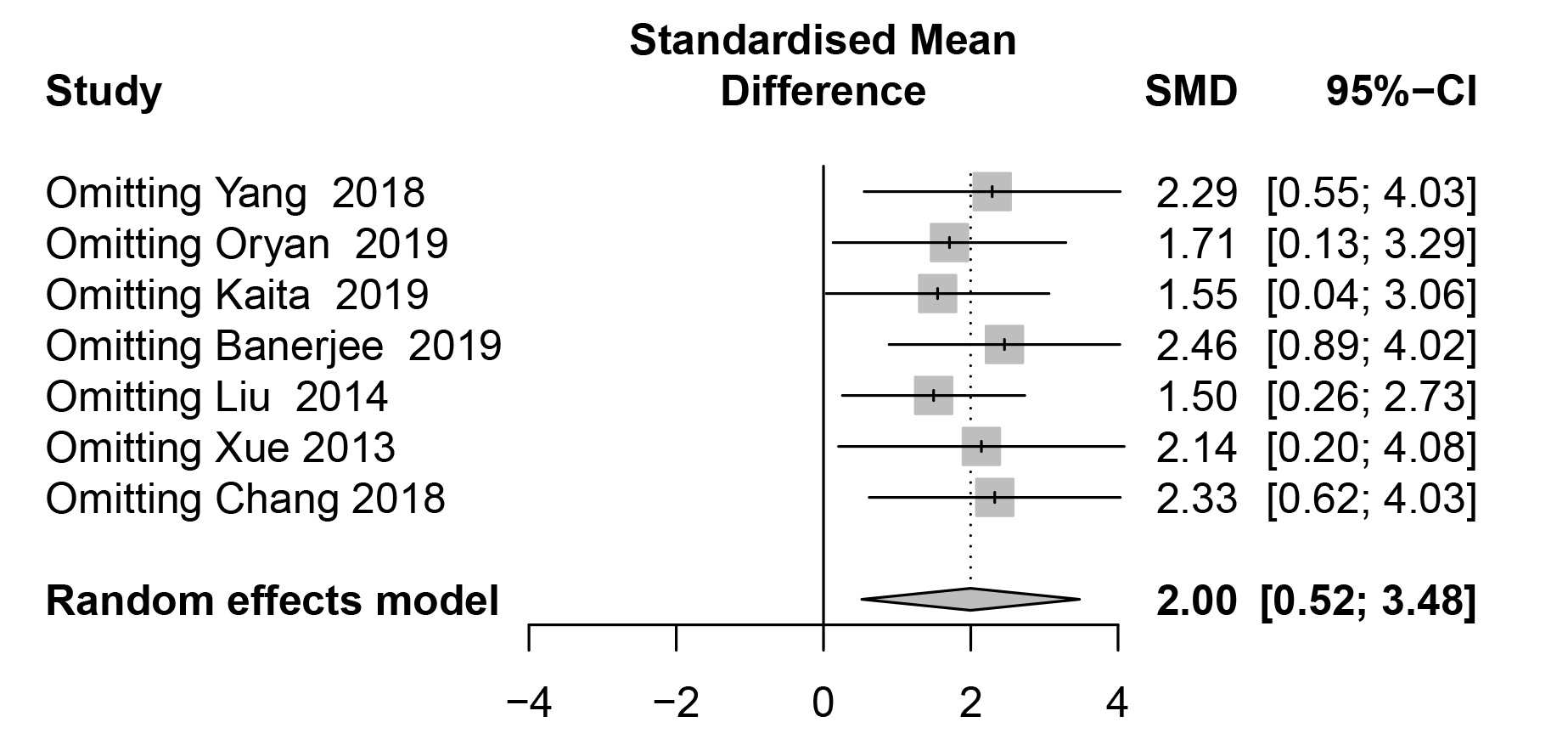

Supplement: Supplementary file 4 — Additional file 4. Leave-one-out meta-analysis of the closure rate on the 14th day. The leave-one-out meta-analysis calculated with random-effects models showed that the final closure rate was so stable that it could be significantly impacted by a single study. [file 13287_2020_1879_MOESM4_ESM.tif]

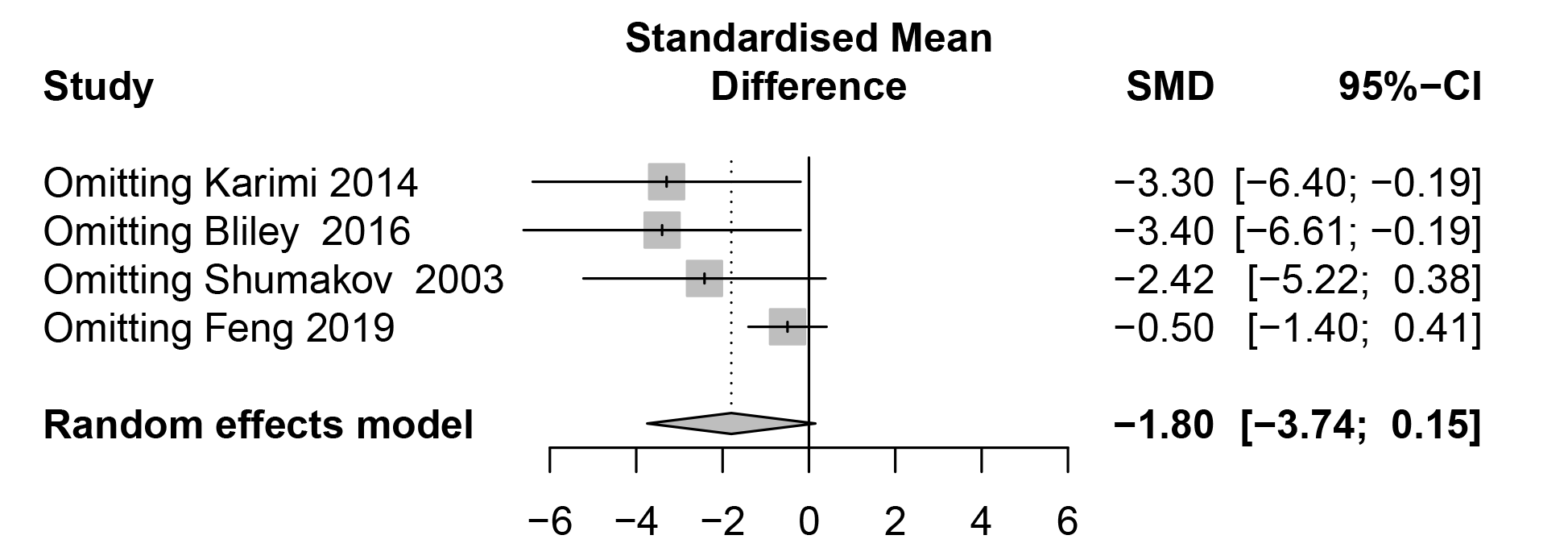

Supplement: Supplementary file 5 — Additional file 5. Leave-one-out meta-analysis of the wound area at the 1st week. The leave-one-out meta-analysis calculated with random-effects models showed that the final result of the wound area could be easily affected by two studies conducted by Karimi and Bliley. [file 13287_2020_1879_MOESM5_ESM.tif]

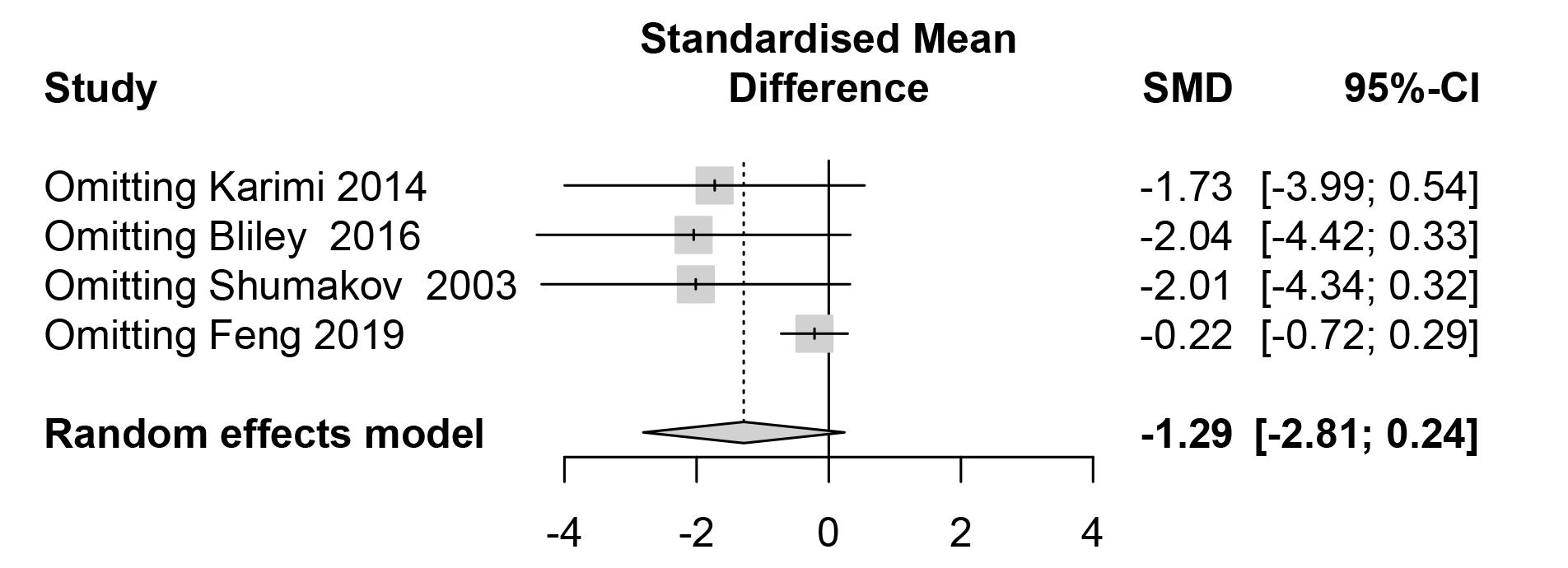

Supplement: Supplementary file 6 — Additional file 6. Leave-one-out meta-analysis of the wound area at the 2nd week. Leave-one-out meta-analysis calculated with random-effects models showed that the pooled outcome of the wound area was not significantly changed by a single article. [file 13287_2020_1879_MOESM6_ESM.tif]

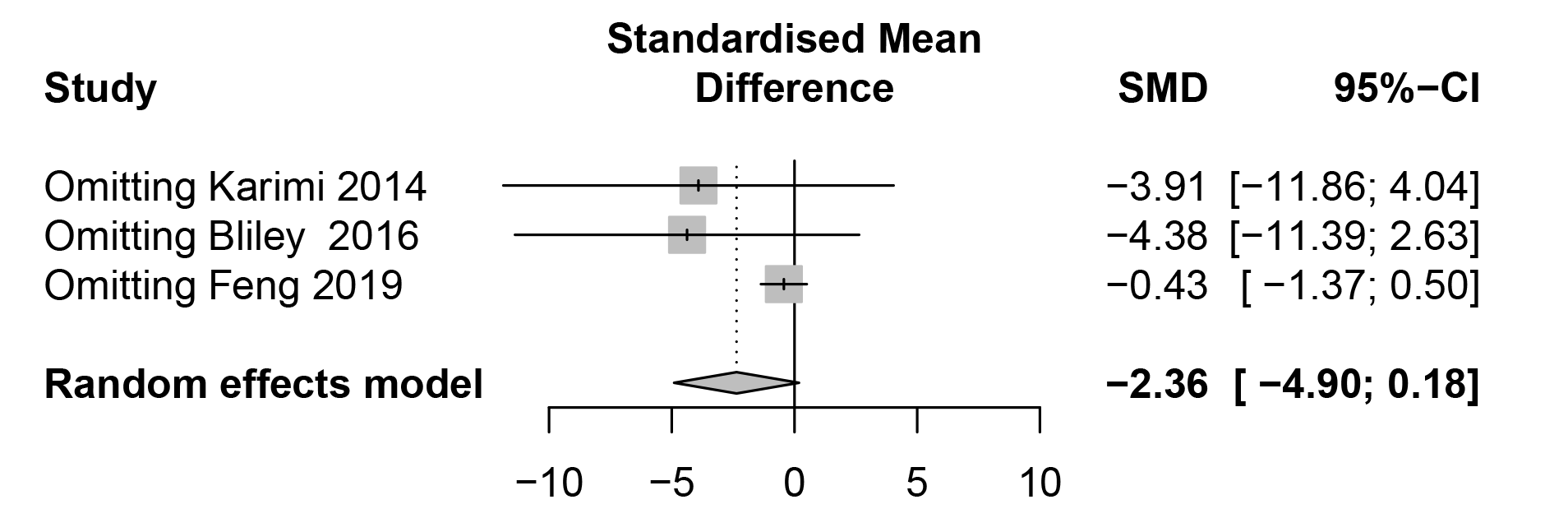

Supplement: Supplementary file 7 — Additional file 7. Leave-one-out meta-analysis of the wound area at the 3rd week. Leave-one-out meta-analysis calculated with random-effects models showed that the pooled outcome of the wound area was not significantly changed by a single article. [file 13287_2020_1879_MOESM7_ESM.tif]
